# Novel Strain-Based Triple Inactivated Vaccine Confers Rapid Neutralizing Immunity to Feline Multisystemic Pathogens With Two-Dose Regimen

**DOI:** 10.1155/tbed/9642624

**Published:** 2025-08-07

**Authors:** Hongchao Wu, Xinyi Li, Ningning Cui, Yujiao Cao, Caihong Liu, Hangtian Ding, Yalei Chen, Yihang Yang, Xiao Chen, Xiaorui Su, Suling Zhang, Lulu Wang, Xiangfeng Xi, Jinhao Bi, Yuxiu Liu, Kegong Tian

**Affiliations:** ^1^National Research Center for Veterinary Medicine, Luoyang, China; ^2^College of Veterinary Medicine, Henan Agricultural University, Zhengzhou, China; ^3^Center for Infectious Disease Research, Westlake University, Hangzhou, China; ^4^Huiizon Co. Ltd., Luoyang, China

**Keywords:** FCV, feline triple vaccine, FHV-1, FPV, Meowonder

## Abstract

The rapid global increase in companion animal populations and the rising risks of zoonotic diseases necessitate urgent advancements in veterinary vaccines. In China, over 100 million domestic cats are vulnerable to three deadly pathogens: feline calicivirus (FCV), feline herpesvirus type 1 (FHV-1), and feline panleukopenia virus (FPV). Existing trivalent vaccines face challenges, such as antigenic mismatches, supply chain inefficiencies, and delayed regional adaptability, highlighting the need for localized solutions. In response, we present Meowonder, the first fully indigenous triple feline vaccine developed using contemporary Chinese isolates of FCV, FHV-1, and FPV. Through inactivation and formulation with a proprietary adjuvant, Meowonder has achieved superior safety and immunogenicity and has been approved for the Chinese market. In controlled trials, two doses of Meowonder resulted in robust neutralizing antibody responses, surpassing commercial vaccines in preventing clinical symptoms such as oral ulcers, respiratory distress, and tissue damage after exposure. Clinical studies involving 1818 cats confirmed the efficacy of the two-dose regimen across various ages and regions, with no significant benefit from a third dose. Significantly, Meowonder reduced viral shedding and maintained healthy leukocyte levels following FPV exposure, which is crucial for environments with multiple cats. Histopathological analyses indicated complete protection against FCV-associated mucosal necrosis, FPV-induced intestinal lesions, and FHV-1-driven respiratory damage. By aligning vaccine antigens with circulating strains, Meowonder addresses regional virological diversity and sets a new standard for feline immunization. It offers a scalable solution to enhance the health of companion animals in China and beyond.

## 1. Introduction

The management of companion animal health has become a critical area within veterinary science, largely due to the rapid increase in global pet populations and a growing awareness of zoonotic disease risks. In China alone, there are over 100 million domestic cats [1] that face ongoing threats from three highly contagious and deadly pathogens: feline calicivirus (FCV), feline herpesvirus type 1 (FHV-1), and feline panleukopenia virus (FPV).

Despite the availability of trivalent vaccines worldwide, many regions experience significant gaps in vaccine accessibility and effectiveness, primarily due to reliance on nonlocalized formulations. Issues such as fragile supply chains, antigenic mismatches with circulating strains, and delayed responses to regional outbreaks highlight the urgent need for new vaccines that are specifically developed based on the evolving virological landscape [2–4].

The antigenic divergence between vaccine prototypes and circulating strains poses a major challenge to vaccine efficacy. Molecular surveillance indicates that FCV field isolates in China show an average amino acid variation of up to 13.95% in the VP1 capsid proteins compared to historical vaccine strains [5]. In contrast, the glycoprotein B sequence of FHV-1 initially shows high conservation, with only partial amino acid mutations occurring later [6]. These differences complicate cross-protection [7], particularly against FCV-associated virulent systemic disease (VSD) and FHV-1 ocular/respiratory syndromes, which pose infection risks of 53.33% [8] and 55.26% [9] for unvaccinated cats, respectively. Conventional methods that use laboratory-attenuated strains fail to keep pace with the rapid evolution of RNA viruses like FCV, where quasispecies dynamics contribute to immune evasion. To address this gap, we present the first systematic development of a fully indigenous triple feline vaccine called Meowonder. This vaccine utilizes contemporary Chinese isolates of FCV, FHV-1, and FPV, fulfilling both the scientific need for strain-matched immunogens and the diversity of veterinary biologics. Compared to traditional vaccines currently available, Meowonder can provide protection with just two doses and stimulate excellent levels of neutralizing antibodies in clinical cohorts.

## 2. Materials and Methods

### 2.1. Biosafety and Animal Ethics Statement

The isolation, cultivation, amplification, and inactivation validation of the virus in this project were all completed in a Biosafety Level 2 (BSL-2) laboratory. Animal experiments were conducted in an Animal Biosafety Level 2 (ABSL-2) facility and were approved by the Animal Experiment Ethics Committee of the National Veterinary Research Center (Approval Number: 202307001). During the study, each cat was housed individually in a cage, with different groups housed in separate areas. All animals had access to adequate food and water and received veterinary care. This study strictly adhered to the Guidelines for the Management and Use of Laboratory Animals established by the People's Republic of China.

### 2.2. Cells, Viruses, Antibodies, and Vaccine Preparation

Feline kidney cells (F81 cells) were maintained in Roswell Park Memorial Institute (RPMI) 1640 medium (Gibco, 61870036), supplemented with 8% fetal bovine serum (Cegrogen Biotech, A0500), 100 U/mL penicillin, and 0.1 mg/mL streptomycin (Gibco, 15140122) at 37°C with 5% CO_2_. The FPV, FCV, and FHV-1 were isolated from anal, eyelid, and nasal swab suspensions of virus-infected cats in Henan Province in 2016. The viruses were isolated as previously described [10]. Briefly, the anal, eyelid, and nasal swab suspensions were filtered and added to F81 cells. When cytopathic effects (CPEs) were observed in 80% of the cells, the cultures were harvested, and the resultant virus stocks were stored at −80°C and designated as FPV strain 708, FCV strain 60, and FHV-1 strain 64. The inactivated vaccine was prepared using the FPV, FCV, and FHV-1. Inactivation of viruses and the validation methods were as previously described [11]. Briefly, the viral solution was centrifuged at 5000 rpm/min at 4°C for 30 min to remove cellular debris. The inactivating agent β-propiolactone (SERVA, 33672.51) was added at a ratio of 1:1000, mixed by inverting the tube, and then incubated at 4°C for 24 h, followed by incubation at 37°C for 2 h to hydrolyze the β-propiolactone. The inactivated viral solution was inoculated into F81 cells and continuously cultured for three generations without showing any CPEs, indicating complete inactivation. AF488 (A32723) or HRP (31460)-conjugated anti-mouse IgG (H + L) antibody was purchased from Invitrogen. The specific mouse monoclonal antibodies against the three viruses were all prepared in-house. Anti-FPV mAb 4B1 targets the VP2 protein, anti-FCV mAb 5G5 targets the Cap protein, and anti-FHV-1 mAb 5H8 targets the gD protein. These antibodies are stored in our laboratory (National Research Center for Veterinary Medicine).

### 2.3. Immunofluorescence Analysis

The F81 cells were seeded in a 96-well plate with cell-crawling sheets in advance to infect the virus (MOI = 0.1). After 96–120 h, the cells were fixed with 4% paraformaldehyde for 20 min at 4°C, blocked with 5% BSA solution overnight at 4°C. After a 400-fold dilution with self-made mouse monoclonal antibodies of different viruses, the cells were incubated at 37°C for 1 h. The Alexa Fluor 488-conjugated secondary antibody was then diluted 2000-fold and incubated at 37°C for 45 min.

### 2.4. Transmission Electron Microscopy (TEM) Analysis

The purified inactivated virus solution was diluted 80 times with PBS and incubated with a 200 mesh copper-plated grid for 1 h at room temperature. Then, excess liquid was blotted from the edges of the mesh, after which the sample was negatively stained with 2% phosphotungstic acid solution (PTA) for 2 min and then air-dried. The observation was performed using a transmission electron microscope (Hitachi TEM system, HITACHI) at an accelerating voltage of 80 keV and a magnification of 40,000 times.

### 2.5. Immunization and Challenge

**Figure 1:**
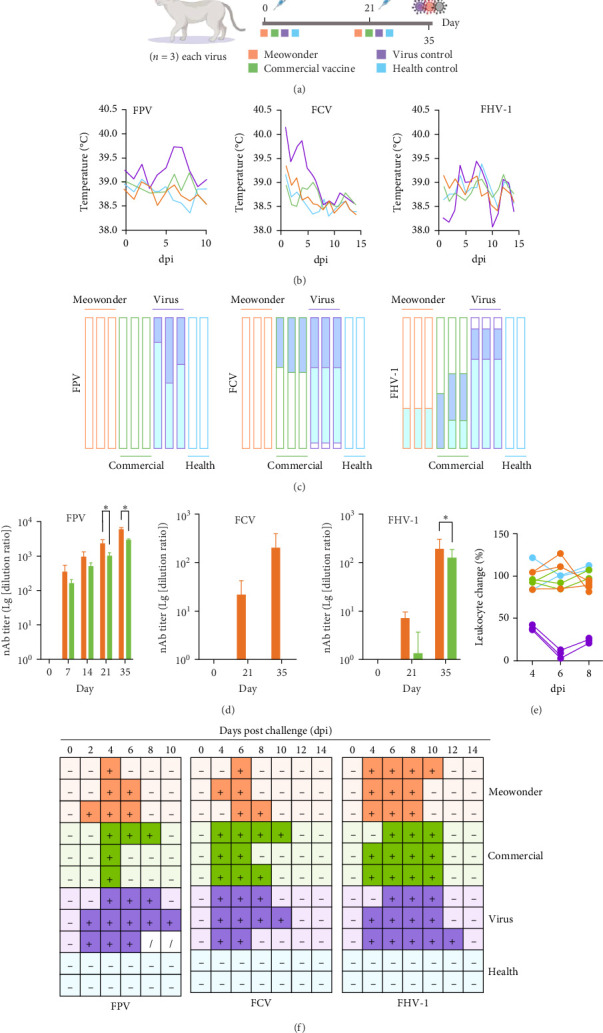
Trial immunological evaluation of Meowonder vaccine. (A) Immunization strategy for the cat animal model. The vaccination was divided into four groups, with the healthy cat control group, which was not infected with any virus at day 35. The viral infection control group, receiving PBS injections at day 0 and 21 and being infected with the virus at day 35. Except for the health group (*n* = 2), *n* = 3 for each group. (B) The body temperature change curve after viral infection. The *x*-axis represents the continuous monitoring time after infection. (C) Evaluation of clinical symptoms in cats after viral infection. Each column represents the clinical score of a cat. Healthy status is indicated by a white-filled rectangle, abnormal status by light blue, and diseased status by dark blue. (D) The serum neutralizing antibody titer during the immune period. The *x*-axis represents the time of vaccination, and the *y*-axis represents the serum dilution, presented on a logarithmic scale. The data were analyzed using two-way ANOVA, with multiple comparisons performed at each time point across groups. (E) Changes in the white blood cell (WBC) count of peripheral blood in cats of the FPV challenge group. (F) Viral nucleic acid detection in fecal swabs (FPV) or eyelid swabs after viral infection. Each row represents an animal, “+” indicates positive, “−” indicates negative, and “/” indicates the animal's death. “*⁣*^*∗*^” indicates statistically significant at *p* < 0.05.

Eleven healthy cats (Chinese domestic cat and nonpedigree indigenous cat), free from FCV, FPV, FHV-1, and feline coronavirus (FCoV) infection, were randomly divided into four groups: (1) Meowonder group (*n* = 3); (2) commercial vaccine group (*n* = 3); (3) virus group (*n* = 3); (4) health group (*n* = 2). Since the first vaccination typically occurs when cats are young, kittens aged 2–3 months weighing over 1 kg were selected. The virus group served as the viral infection control and received PBS throughout the study, while the health group served as the healthy control and received PBS but was not subjected to viral challenge. The challenge doses were FPV 10^5.5^ FAID_50_, FCV 10^7.0^ TCID_50_, and FHV-1 10^4.5^ TCID_50_. Three parallel cohorts were established for the aforementioned groups to evaluate vaccine efficacy following three types of viral challenges, resulting in a total of 33 cats used in the study. Each cat was housed in individual cages, with different groups separated into distinct spaces. According to the immunization schedule (Figure 1A), the cats were subcutaneously vaccinated on days 0 and 21. On day 35 postfirst immunization, each cat was infected with FPV, FCV, or FHV-1. Clinical symptoms and body temperature were monitored daily, and venous blood was collected on days 0, 21, and 35 to assess virus-neutralizing (VN) antibody levels. For the FPV cohort, anticoagulated whole blood was collected via the jugular or cephalic vein at 4, 6, and 8 days postinfection following the viral challenge. According to the manufacturer's instructions, the total white blood cell (WBC) count in the blood was measured using a Veterinary Automatic Hematology Analyzer (Mindray, BC-5000Vet).

### 2.6. Neutralization Assay

The serum was analyzed for FPV, FCV, and FHV-1 VN antibodies by heat inactivation at 56°C for 30 min, followed by a twofold serial dilution from 1:2 to 1:256, as per the previous method [12]. A 200 TCID_50_ dose of FPV, FCV, or FHV-1 was mixed with the serum sample in a 1:1 volume ratio, incubated at 37°C for 1 h, and subsequently added to F81 cells (2 × 10^4^ cells/100 μL). After a 4-day incubation at 37°C with 5% CO_2_, VN antibody titers were determined by CPE and expressed as the reciprocal of the highest dilution that inhibited infection of the F81 cells in 50% of the culture wells.

### 2.7. Clinical Signs and Histological Analysis

Clinical scores were obtained by trained observers who were blinded to the each groups from day 0 to day 14 post-FPV, FCV, and FHV-1 challenge, as described in previous studies [13, 14]. Two observers recorded symptoms for 30 min each morning for each group. Clinical symptoms included fever, ocular discharge, sneezing, and nasal discharge, with specific evaluation criteria and symptom severity grading detailed in the Supporting Information 1: Table S1. Fourteen days postviral challenge, duodenal, jejunal, ileal, eyelid, nasal turbinate, tracheal, and lung tissues were collected from the cats. All tissue samples were fixed in 4% paraformaldehyde, embedded in paraffin, sectioned, and stained with hematoxylin and eosin (H&E). Mouse monoclonal antibodies specific to each virus were used for immunohistochemical (IHC) analysis.

### 2.8. Viral Shedding

After the challenge, fecal swabs (FPV) or eyelid swabs (FCV and FHV-1) were tested every 2 days for FPV, FCV, and FHV-1 DNA. Swabs were collected in 1 mL of PBS, and viral nucleic acid was extracted from 200 μL of the swab samples using the Viral Nucleic Acid Extraction Kit (Geneaid Biotech Ltd., VR300). The primers for the PCR test were previously described [12, 15]. The reaction conditions were as follows: predenaturation at 95°C for 3 min, denaturation at 95°C for 30 s, annealing at 58°C for 30 s, and elongation at 72°C for 30 s, repeated for a total of 30 cycles. Amplicons were detected on 1.5% agarose gels.

### 2.9. Clinical Collection Criteria and Methods

A standard two-dose vaccination schedule was implemented in clinical veterinary hospital settings, with doses administered at 3–4 week intervals. While most animals received two doses according to protocol, some received a third dose based on individual veterinarians' clinical practice. Blood samples were collected 3–4 weeks following the final vaccination. The serum was separated, and neutralizing antibody titers were determined.

### 2.10. Statistical Analysis

Statistical analysis was performed using GraphPad Prism software (version 9.02). When analyzing the statistical differences between two data groups, the sample's hypothesis type was judged according to the homogeneity of variance of the data (equal variance/heteroscedasticity). The interaction between the mean values of each dataset was considered in the case of three or more datasets. Single response variables were analyzed using one-way ANOVA. Two-response variables were analyzed using two-way ANOVA. Differences with *p*-values less than 0.05 were considered statistically significant.

## 3. Results

### 3.1. Isolated Highly Pathogenic FPV, FCV, and FHV-1 as Candidate Vaccines

**Figure 2:**
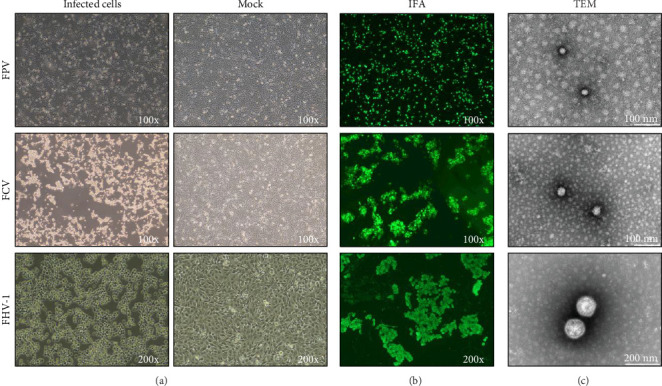
Virological identification of clinical isolates. (A) The three viruses were inoculated into the F81 cell line at a confluency of 90% with a multiplicity of infection (MOI) of 0.1. The cytopathic effect (CPE) was observed after 96–120 h of incubation at 37°C and 5% CO_2_. The cells in the right column serve as uninfected controls. (B) F81 cells were subjected to IFA identification 72 h postviral infection. The initial antibodies for IFA were prepared from specific mouse monoclonal antibodies against the three inactivated viruses (1:400), followed by incubation with mouse secondary antibodies conjugated to AF488 (1:2000). Observations were made under a fluorescence microscope. (C) The morphological characteristics of viruses were analyzed by TEM.

Since 2016, we have collected clinical samples of viral infections from veterinary clinics across China. Secretions from anal, eyelid, and nasal swabs collected from cats were inoculated onto F81 cells to isolate the viruses. After inoculation, these cells were cultured, and the emergence of CPEs was consistently observed (Figure 2A). Plaque purification was performed on F81 cells exhibiting CPE to obtain viral strains from individual clones. Following expansion, the cultures were reinoculated into fresh F81 cells, and immunofluorescence identification was conducted using mouse monoclonal antibodies (Figure 2B). This process enabled the isolation of wild-type FPV strain 708, FCV strain 60, and FHV-1 strain 64 from the clinical specimens. After inactivation, these viruses were concentrated and purified, maintaining their complete viral particle morphology (Figure 2C). A self-developed, painless, and low-sensitivity adjuvant was utilized to prepare a trivalent vaccine for cats that targets significant health threats and has been upgraded to the new vaccine named Meowonder. The adjuvant is an aqueous adjuvant composed of plant saponins and natural phospholipids, enhancing antigens' immunogenicity while avoiding the side effects of oil-based adjuvants. Since the immunogen is a prevalent strain of a highly pathogenic virus, inactivated, carefully integrated immunogenicity and safety considerations were implemented in the vaccine design.

### 3.2. The Meowonder Showed Excellent Immune Protection in Cats

Cats were used as an animal model to assess the preventive effects of Meowonder, and a systematic evaluation was conducted. As illustrated in Figure 1A, the cats were divided into four groups: the Meowonder group, the commercial vaccine group, the healthy group, and the virus group. According to our immunization schedule, the cats received vaccinations on day 0 and day 21, followed by a viral challenge on day 35. Three parallel cohorts were established to evaluate the vaccine's protective efficacy against each virus individually.

Following the viral challenge, the virus control groups for the FPV, FCV, and FHV-1 cohorts exhibited fever symptoms (greater than 39.5°C). FCV and FPV virus control were significantly higher than those observed in the healthy control (Figure 1B). The body temperature changes in both vaccine groups aligned with those in the healthy control, showing no statistically significant differences (Supporting Information 1: Table S1). Notably, after FCV infection, the commercial vaccine group displayed symptoms of oral ulcers and sticky eyelid secretions, similar to the virus control. In contrast, these symptoms were not evident in the Meowonder group (Figure 1C). After infection with FHV-1, the Meowonder group experienced only mild sneezing symptoms, while the commercial vaccine group showed significantly reduced activity and sticky eyelid discharge. Supporting Information 2 can find all clinical symptoms and scoring criteria following the viral challenge. Although clinical observations suggest that Meowonder has the potential as a two-dose vaccine, measuring neutralizing antibody levels remains the gold standard for evaluating vaccine efficacy.

As shown in Figure 1D, the Meowonder group rapidly generated neutralizing antibodies after the initial immunization. Although all neutralizing antibody titers were higher than those in the commercial vaccine group, statistical differences were observed only at specific time points. To account for fluctuations in antibody levels throughout the immune monitoring period, two-way ANOVA was employed instead of paired *T*-tests to compare each virus at individual time points. These results indicate a more robust neutralizing antibody response in the Meowonder group compared to the commercial vaccine group. Meanwhile, the neutralization assay used the wild-type strain from China. This further indicates that classical commercial vaccines have difficulty in inducing neutralizing antibodies against prevalent strains in China, especially FCV.

In practical vaccine application scenarios, especially for pet breeding and multi-cat households, vaccination is crucial in improving survival rates and impacting viral infections in other animals. The viral nucleic acid in feces or eye secretions was tested after the viral challenge to determine if vaccination could completely block viral shedding through the digestive tract or respiratory tract. In conjunction with the pathogenic characteristics of FPV, tests for key biological markers were also conducted. As shown in Figure 1E, following the FPV challenge, the vaccinated group maintained healthy WBC levels. Figure 1F indicates that the Meowonder group cleared the shedding phenomenon earlier than the commercial vaccine group. These results suggest that the two-dose Meowonder vaccine offers more protective benefits.

### 3.3. The Meowonder Vaccine Protects Cats From Tissue Injury

**Figure 3:**
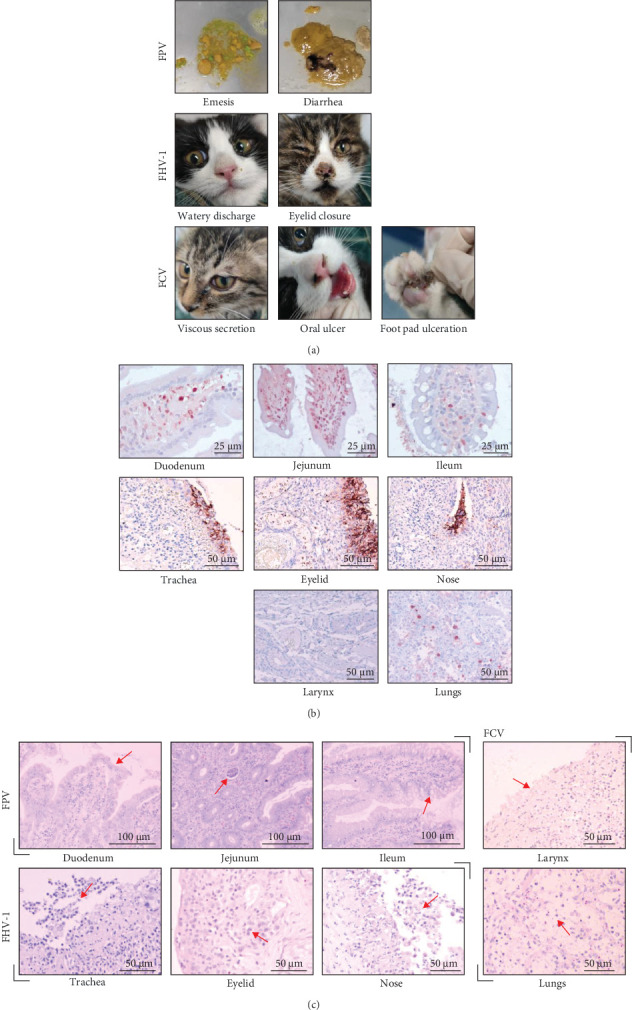
Clinical symptoms and histopathological changes after viral infection. (A) Clinical symptoms exhibited by the mock group after viral challenge in the evaluation of vaccine efficacy. (B) IHC analysis of key tissues affected by viral infection. The first row represents the FPV challenge group, the second row FHV-1, and the third row FCV. (C) Histological lesions shown in H&E stained sections. Red arrows indicate typical lesions.

After the study, the cat was euthanized to collect tissue samples and assess the pathological changes in its tissues. During the postmortem examination, no significant lesions were observed in the cat. Typical clinical symptoms and tissue lesions were primarily recorded in the viral control (Figure 3A), while the Meowonder vaccine had no tissue lesions.

IHC results revealed that the lesions induced by FPV were limited to the duodenum, jejunum, and ileum (Figure 3B). The histopathological changes included the separation and shedding of duodenal mucosal epithelial cells from the lamina propria; necrotic and shed epithelial cells were visible in the jejunal crypts, along with an increase in the number of goblet cells in the ileal mucosal epithelial tissue (Figure 3C, red arrows). FCV infection, the virus was primarily concentrated in the pharynx and lungs (Figure 3B). H&E staining revealed extensive neutrophilic necrosis of the pharyngeal mucosa, with macrophage and neutrophil infiltration in the alveolar spaces (Figure 3C, red arrows). In the case of FHV-1 infection, lesions were limited to the nasal cavity, bronchi, and eyelids (Figure 3B). These lesions were characterized by significant areas of necrotic sloughing of the nasal mucosal epithelial cells, substantial inflammatory cell infiltration in the lamina propria, necrosis and sloughing of local tracheal mucosal epithelial cells, and extensive inflammatory infiltrate in the eyelid mucosa (Figure 3C, red arrows). These findings indicate that the Meowonder vaccine can protect tissues from damage caused by FPV, FCV, and FHV-1 infections.

### 3.4. Two Doses of Meowonder Sufficiently Induce a Strong Neutralizing Response in Clinical Cohorts

**Figure 4:**
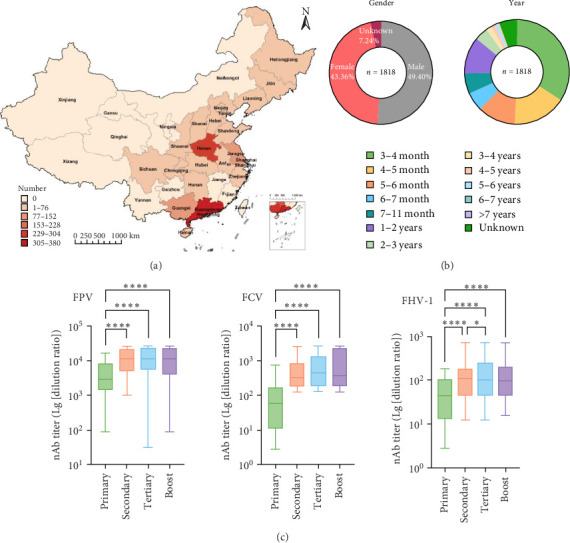
Meowonder vaccine clinical cohort investigation. (A) Provinces covered by the samples. (B) The gender and age composition of the serum samples. (C) Neutralizing antibody levels against different viruses. The *x*-axis represents the number of vaccine doses, sequentially indicating one-dose, two doses, three doses, and one booster. “*⁣*^*∗∗∗∗*^“ indicates statistically significant at *p* < 0.0001.

By December 31, 2024, we had collected 2168 serum samples from individuals vaccinated with Meowonder in the pet clinic, of which 1818 were valid for neutralization experiments, representing 21 provinces in China (Figure 4A). The samples adequately cover various stages of a cat's age, primarily focusing on kittens aged 3–6 months for initial immunization or before sexual maturity (Figure 4B). The background includes 26 pet breeds, such as the Chinese Domestic, British Shorthair, and American Shorthair. Figure 4C shows no statistically significant difference in the neutralizing antibody titers for FPV and FCV between cats receiving two and three vaccinations. This finding aligns with previous results observed in ABSL-2 studies. Notably, the neutralizing antibodies against FHV-1 increased after the third immunization. Although Meowonder advocates a two-dose vaccination regimen, small number of clinicians in actual practice often prefer administering three doses. The three-dose regimen included 151 valid data points for statistical analysis, while the two-dose regimen included 1390. This might be the reason for the observed statistical difference. In 1818 samples, the average neutralizing antibody titer for FPV was 1:12,642.84; FCV = 1:726.32; FHV-1 = 1:146.58. For adult cats aged 1 year and above with a history of immunization, the booster vaccination of Meowonder resulted in significantly higher levels of neutralizing antibodies than the average value. The average neutralizing titers for FCV in cats vaccinated twice were 1:750.76, and in those vaccinated three times, it was 1:859.51. This indicates that while a two-dose regimen can produce high antibody levels against FCV, a three-dose regimen further enhances these antibody levels in cats. After a single dose, the average neutralizing titer for FHV-1 was 1:43.33; two doses = 1:145.30, three doses = 1:146.58. These results confirm that the immunization strategy for Meowonder, which requires only two doses, is superior to commercially available vaccines. The above results indicated that the Meowonder vaccine can induce an excellent immune response with only two immunization doses, regardless of the cats' regions, sexes, or ages.

## 4. Discussion

The development of the Meowonder triple feline vaccine represents a critical advancement in addressing region-specific challenges in companion animal health. By aligning vaccine antigens with circulating strains in China, this study demonstrates that localized immunogen design can overcome the limitations of traditional vaccines, particularly in rapidly evolving RNA viruses like FCV. Beyond its scientific merits, the practical implications of Meowonder extend to diverse real-world scenarios where feline health management is paramount, including pet breeding facilities, multi-cat households, and animal shelters.

In commercial catteries and shelters, overcrowding amplifies the risk of pathogen transmission. For instance, FCV and FHV-1, which spread via respiratory droplets and fomites, can infect up to 64.3% of unvaccinated cats in such settings [16]. Traditional vaccines often fail to prevent outbreaks due to antigenic mismatches [17, 18], as evidenced by the 53.33% FCV infection rate in unvaccinated cats [8]. Meowonder's two-dose regimen, achieving neutralizing titers of 1:726.32 against FCV and 1:146.58 against FHV-1 within 35 days (Figure 4C), offers a rapid and reliable solution. Its efficacy in blocking viral shedding (Figure 1F) is particularly crucial for facilities where fecal-oral transmission of FPV could decimate kitten populations. By reducing subclinical shedding, the vaccine not only protects individual animals but also disrupts transmission chains, a feature critical for maintaining biosecurity in high-density environments.

For urban pet owners, vaccine compliance often hinges on logistical simplicity. The standard three-dose protocol for kittens, coupled with annual boosters, poses challenges for busy households. Meowonder's two-dose priming schedule, validated across 1818 clinical cohorts (Figure 4A), simplifies immunization without compromising efficacy. This regimen also minimizes stress for young cats, as fewer veterinary visits reduce exposure to clinic-associated pathogens. Furthermore, the vaccine's painless adjuvant addresses a common owner concern: The owner is hesitant about the possible discomfort that may occur after the pet is vaccinated [19]. By enhancing both compliance and welfare, Meowonder aligns with the growing demand for feline healthcare that prioritizes quality of life.

While Meowonder shows promise, its implementation requires addressing vaccine hesitancy through public education, especially regarding the administration of a brand-new rather than a traditional brand of vaccine. Long-term and positive clinical feedback is needed to make the public accept Meowonder. In clinical cohort analysis, we found that the neutralizing titers of FPV in 44 sera from two-dose immunizations were higher than 1:20,000, while the levels of FHV or FCV were extremely low. To this end, we conducted follow-up interviews one by one and found that these cats invariably had a history of FPV exposure before or during the administration of Meowonder. These cats produced extremely high neutralizing antibodies under the multiple stimulations of vaccination and wild virus strains (Supporting Information 3: Figure S1). In addition, the cohort data included nine sera with lower neutralizing antibody titers (eight from two doses and one from three doses), with the FPV neutralizing titer ranging from 1:91.2 to 1:724.4, FCV from 1:10 to 1:162.2, and FHV-1 from 1:10 to 1:15.8. These results may indicate that a certain proportion of the samples in the cohort have an impaired immune system or are nonpathogen susceptible (Figure S1).

In conclusion, Meowonder exemplifies how regionally tailored vaccines can transform feline health management across ecological and socioeconomic gradients. By bridging the gap between virological surveillance and applied immunology, this work provides a blueprint for combating antigenic drift in companion animal pathogens globally.

## Data Availability

The data supporting this study's findings are available from the corresponding author upon reasonable request.

## Supporting Information

Supporting Information 1 Statistical analysis of temperature data (Table S1). Statistical comparison of body temperature changes among groups when evaluating the efficacy of Meowonder.

Supporting Information 2 Scoring details of clinical symptoms. The specific records of abnormal clinical symptoms exhibited by the groups of cats when evaluating the efficacy of the Meowonder vaccine.

Supporting Information 3 Distinctive data in the clinical cohort (Figure S1). Display of individual data with abnormal neutralizing antibody levels in the clinical cohort data sample.
