# Supplementary material for: Novel Strain-Based Triple Inactivated Vaccine Confers Rapid Neutralizing Immunity to Feline Multisystemic Pathogens With Two-Dose Regimen
Source: Transbound Emerg Dis. 2025 Aug 7;2025:9642624. doi: 10.1155/tbed/9642624 (PMC12352998; doi:10.1155/tbed/9642624)
Supplement: Supporting Information 1 — Statistical analysis of temperature data (Table S1). Statistical comparison of body temperature changes among groups when evaluating the efficacy of Meowonder. [file 9642624.f1.docx]

**Table S1:** **Statistical analysis of temperature data**

| Virus | Tukey's multiple comparisons test | Summary | Adjusted P Value |
| --- | --- | --- | --- |
| FPV | Meowonder^TM^ vs. COM'I Vaccine | ns | 0.1736 |
|  | Meowonder^TM^ vs. Virus control | *** | 0.0009 |
|  | Meowonder^TM^ vs. Health control | ns | 0.8609 |
|  | COM'I Vaccine vs. Virus control | ** | 0.0047 |
|  | COM'I Vaccine vs. Health control | ns | 0.8298 |
|  | Virus control vs. Health control | * | 0.0160 |
| FCV | Meowonder^TM^ vs. COM'I Vaccine | ns | 0.9631 |
|  | Meowonder^TM^ vs. Virus control | ** | 0.0029 |
|  | Meowonder^TM^ vs. Health control | ns | 0.3086 |
|  | COM'I Vaccine vs. Virus control | ns | 0.0532 |
|  | COM'I Vaccine vs. Health control | ns | 0.3980 |
|  | Virus control vs. Health control | ** | 0.0026 |
| FHV-1 | Meowonder^TM^ vs. COM'I Vaccine | ns | 0.9999 |
|  | Meowonder^TM^ vs. Virus control | ns | 0.9544 |
|  | Meowonder^TM^ vs. Health control | ns | 0.9740 |
|  | COM'I Vaccine vs. Virus control | ns | 0.9324 |
|  | COM'I Vaccine vs. Health control | ns | 0.9673 |
|  | Virus control vs. Health control | ns | 0.7212 |
