# Supplementary material for: Novel Strain-Based Triple Inactivated Vaccine Confers Rapid Neutralizing Immunity to Feline Multisystemic Pathogens With Two-Dose Regimen
Source: Transbound Emerg Dis. 2025 Aug 7;2025:9642624. doi: 10.1155/tbed/9642624 (PMC12352998; doi:10.1155/tbed/9642624)
Supplement: Supporting Information 3 — Distinctive data in the clinical cohort (Figure S1). Display of individual data with abnormal neutralizing antibody levels in the clinical cohort data sample. [file 9642624.f3.docx]

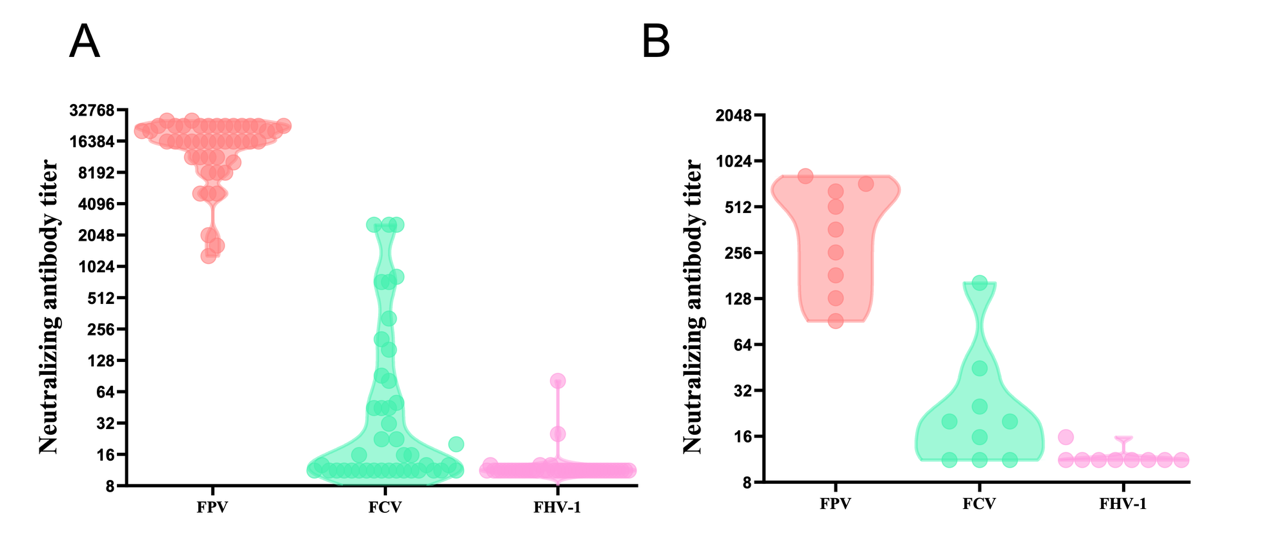


**Figure S1:** **Distinctive data in the clinical cohort**

（A）Serum data of individuals with a history of FPV exposure before or during vaccination.

（B）The immune system may be deficient or not sensitive to the pathogen.
